# 3D-Printed Self-Assembling Helical Models for Exploring Viral Capsid Structures

**DOI:** 10.3390/biomimetics9120763

**Published:** 2024-12-16

**Authors:** Donald Plante, Keegan Unzen, John R. Jungck

**Affiliations:** 1 Department of Applied Engineering & Sciences, University of New Hampshire at Manchester, Manchester, NH 03101, USA; donald.plante@unh.edu (D.P.);; 2 Departments of Biological Sciences and Mathematical Sciences, University of Delaware, Newark, DE 19716, USA

**Keywords:** self-assembly, 3D-printing, 4D-printing, helix, helical, virus, TMV, capsid, capsomere

## Abstract

This work presents a novel application of additive manufacturing in the design of self-assembling helical viral capsids using 3D-printed components. Expanding on prior work with 3D-printed self-assembling spherical capsids, we developed helical models that integrate geometric parameters and magnetic interactions to mimic key features of the assembly process of helical viral capsids. Using dual-helix phyllotactic patterns and simplified electrostatic simulations, these models consistently self-assemble into a cylinder, providing unique insights into the structural organization and stability of helical capsids. This accessible 3D-printed approach demonstrates the potential of additive manufacturing for research in mesoscale self-assembling models and in the education of complex biological assembly processes, promoting hands-on exploration of viral architecture and self-assembly mechanisms.

## 1. Introduction

Self-assembly, the process by which components spontaneously form organized structures through random local interactions, is a growing field that has applications in various disciplines. These include biological systems like those studied herein in the form of viral capsids, which are the protein shells that encase a virus’s genetic material, as well as other areas like material science and nanotechnology. When Olson’s group at the Scripps Research Institute first developed their self-assembling models based on spherical viral capsids [1,2,3,4,5,6], 3D-printing technology was not as accessible as it is today. However, with the increased adoption of 3D-printers in many homes, secondary schools, and universities today, these self-assembling models can now easily be printed by a much larger audience, allowing for hands-on exploration in both research and education.

Previously, we have expanded on Olson’s work by developing novel 3D-printed spherical self-assembling models based on the geometries of Platonic solids and various Archimedean solids [7,8]. These efforts include a detailed representation of the T = 1 viral capsid model, composed of 60 asymmetric components, shedding light on the assembly dynamics of spherical viruses. Our investigations into spherical viral capsids have been notably enlightening. The use of physical models in our research has underscored their invaluable role in real-time visualization of the assembly process. By observing how our 3D-printed capsomeres interact and come together, we gained crucial insights into the dynamics of self-assembly.

The intricate beauty and significance of helical capsids have long been overshadowed by their icosahedral counterparts, yet they play a crucial role in virology and molecular biology. McDaniel and Wierenga recently reported on icosahedral models of viral capsids and asserted that most viral capsids are icosahedral [9]. While we have worked extensively with self-assembly and self-folding of icosahedral models of viral capsids [7,8], it is important to note that many viruses have helical capsids. Historically, helical capsids were described mathematically before icosahedral models of viral capsids. Helical viruses have received inadequate attention in the self-assembly literature despite their importance on many grounds. The early work on helical viruses is a virtual who’s who [9,10,11,12,13,14,15,16,17,18] of significant crystallographers in molecular biology including several Nobel Laureates: Rosalind Franklin, J. D. Bernal, Donald Caspar, Francis Crick, Aaron Klug, Max Lauffer, James Watson, etc. In medicine, helical viruses such as respiratory syncytial virus (RSV), are notable for their structural study, with related helical viruses like Marburg and vesicular stomatitis virus (VSV) being examined for potential applications in vaccine development, others for drug delivery, and development of new nanobiomaterials [19,20,21,22,23,24,25,26,27,28,29,30,31,32,33,34,35,36,37] The measles virus and the respiratory syncytial virus (RSV), two more commonly known viruses, have interior helical assemblies of protein capsomeres, with RSV gaining heightened attention during the COVID pandemic. In terms of agriculture, helical viruses are associated with the ability of endosymbiotic bacteria to catalyze nitrogen fixation and are necrotic disease agents of many economically important crop species such as potatoes and tobacco [38,39,40,41,42,43,44,45,46,47].

Our transition to helical models stems from the desire to increase our understanding of the self-assembly mechanisms of viral capsids recognizing the diversity of viral architecture and its critical implications for virology. This progression represents a natural extension of our work, deepening our comprehension of viral assembly processes in their varied forms.

In this paper, we present the design and manufacturing of mesoscale, self-assembling helical viral capsid models. Using 3D-printing technology, we demonstrate how physical models can approximate the molecular interactions that guide viral capsid assembly, making these models accessible for both research and educational purposes. By replicating key features of viral architecture, our work provides insight into the dynamics of helical assembly and offers a valuable tool for understanding the formation of viral structures at an accessible scale.

## 2. Overview of Self-Assembling Helical Viral Capsid Models

Mathematical modeling at the mesoscale serves as a powerful tool for visualizing these complex assembly processes, offering valuable insights into the interactions of capsomeres during formation. Similar to most protein–protein interactions, viral capsid assembly results from a combination of hydrophobic, electrostatic, Van der Waals, and hydrogen bonding interactions [32,48,49,50,51,52,53,54,55,56,57]. Weak protein–protein interactions appear to be a common theme in virus assembly. In our models, we simplify these noncovalent bonds using magnetic interactions to represent electrostatic forces, while the geometric design of the 3D-printed components mimics how capsomeres interlock with each other as similarly justified by Olson’s initial work [1,2,3,4,5,6]. Zandi et al. [56] note “Plausibly, the main driving force for capsid assembly is the hydrophobic interaction between apolar patches on the coat proteins, which has to be strong enough to overcome the Coulomb repulsion between the net electrical charge on them. Other types of interaction may also contribute to the stability of virus capsids, of which the most prominent are the complexation with the oppositely charged genome (real or synthetic), and hydrogen bonds or salt bridges involving, e.g., Caspar carboxylate pairs on neighboring coat proteins. The strength of the net attractive interaction between the coat proteins inferred from equilibrium assembly studies are remarkably weak, however, and thought to prevent the growing capsids from becoming kinetically trapped”. Although we have simplified these interactions, our method provides a proof of concept, demonstrating that random local interactions, modeled by magnetic forces between geometrically designed pieces, can lead to the construction of large metastable assemblies, analogous to biological capsids. In particular, we believe that the parallel between entropy driven assembly of proteinaceous capsids [32,53] and the self-assembly (4D-printing) of mesoscale models such as shown herein is a powerful demonstration of random processes generating complex well-organized structures. Furthermore, studies have reported that these dynamic models of self-assembly have enhanced student comprehension of a very counterintuitive process which challenges usual assumptions of design [3,58].

Herein, we illustrate two related helical self-assembling models based on these magnetic and geometric interactions. The first of these models focuses primarily on the geometric shape of the 3D-printed components to facilitate the self-assembly process while also helping to maintain stability of the assembly. In this model, the geometry of each component is specifically designed to fit together similar to puzzle pieces, driving the assembly. The second model, in contrast, shifts the focus of the assembly mechanism towards the attractive forces on the surface of the capsomeres. Specifically, we explored how these charges drive the assembly process by aligning neighboring capsomeres with each other while placing less emphasis on the overall geometry. This model aims to simplify the geometric design, relying instead on charge interactions modeled after the residues of the Tobacco Mosaic Virus (TMV). In this model, positive and negative charge distributions on the surface of the protein capsomeres play the key role in self-assembly, which we will explore in greater detail in Section 4.

In one of the earliest models, Crick and Watson [23] had two rules for self-assembly of viral capsids: (1) Each subunit has identical, equivalent bonding contacts with its neighbors which are able to self-assemble into semi-stable symmetrical arrays. (2) These boding contacts are usually non-covalent so that the process is reversible and error-free. In the case of helical viruses, a rotational symmetry is generated by subunits that curve into a helix as they are thicker at one end and stack upon one another around an axis to produce a hollow tube.

Both of our 3D-printed self-assembling models feature identical subunits with magnetic interactions modeled after the electrostatic charges found on the surface of the capsids of helical viruses, guiding their efficient self-assembly into long helical tubes. In our first model, subunits are stacked in a manner similar to bricks, with their edges aligned along a single right-handed helix. However, our second model incorporates phyllotactic patterns based on Fibonacci numbers, with the edges of the capsids aligned along both right-handed and left-handed helices. For this model we have incorporated 180-degree rotational symmetry about the center of its exterior faces. This feature helps to improve the model’s assembly efficiency by allowing each piece to connect in multiple ways. These mesoscale self-assembling models of helical viral capsids are essential for exploring applications in pharmaceutical design and education.

## 3. Design of the Interlocking Helical Viral Capsid Model

**Figure 1:**
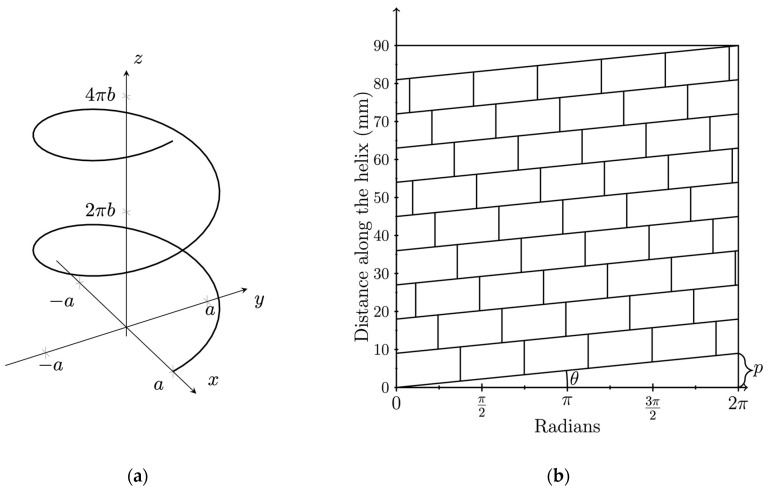
Helix and helical net diagram: (**a**) the helix, as defined by Equation (1), with parameter t varying from 0 to 4π; (**b**) helical net diagram showing the helical angle, θ, where the x-axis is the unwrapped circumference of the helix. The vertical distance between successive layers corresponds to the pitch, p.

The structure of a helix is primarily characterized by two key geometric parameters: the pitch (p) and the helix angle (θ). (Figure 1).

The pitch represents the distance between successive turns of the helix, measured along its main axis, and is critical in determining the tightness of the helix. For example, for the Tobacco Mosaic Virus (TMV), the pitch is 2.29 nm, and the full virus contains 2130 identical capsomeres, with each one consisting of 158 amino acids. The capsomeres form a right-handed helix with 49 subunits per three turns, or approximately 16.3 capsomeres per helical turn. The axial rise per capsomer is 0.14 nm, and the total length of the virus tube is 300 nm.

The helix angle (θ) determines how the capsomeres twist around the central axis, influencing the overall shape. By understanding the relationship between pitch and helix angle, we can accurately model this self-assembly process at the mesoscale, replicating the viral capsid’s behavior in both experimental and theoretical contexts.

To determine the pitch and helical angle of a helix, we used a set of parametric equations that describe its form in three-dimensional space as seen in Figure 1a:

(1) $$x\left(t\right)=\mathrm{acos}\left(t\right),\;y\left(t\right)=\mathrm{asin}\left(t\right),\;z\left(t\right)=bt\;$$

In Equation (1), a denotes the radius of the helix, b is the constant that determines the vertical rise per unit length of the helix, and t is the parametric variable that represents the angle of rotation in radians. From these equations, we can calculate the pitch p of the helix, which is the vertical distance covered as the helix makes one full turn, which is equivalent to a 2π increase in the parametric variable t:

(2) $$p=z\left(t+2\pi \right)-z\left(t\right)=b\left(t+2\pi \right)-bt=2\pi b\;$$

Equation (2) allows us to directly calculate the pitch of the helix from the parameters of the model. The helix angle (θ) reflects the steepness of the helix. This can be visualized by unwrapping the helix onto a grid as scene in Figure 1b. By substituting Equation (2) into this relationship we can see that the helix angle θ is determined by the ratio of the vertical rise b to the radius a, leading to the following:

(3) $$\theta =\arctan\left(\frac{p}{2\pi a}\right)=\arctan\left(\frac{2\pi b}{2\pi a}\right)=\arctan\left(\frac{b}{a}\right)$$

This understanding of helical geometry guided the design of our first 3D-printed model. While we simplified the number of components per turn and the precise helical angle, we maintained the general geometric principles seen in viral capsids to capture the overall behavior of self-assembly.

**Figure 2:**
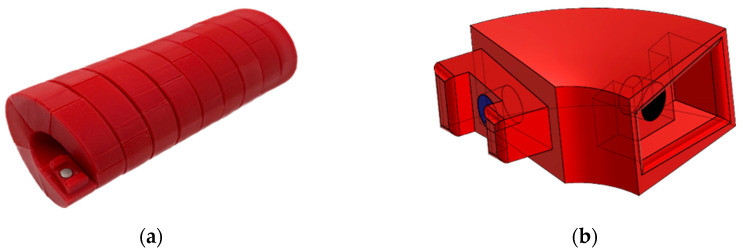
Capsomere and assembled helical tube: (**a**) fully assembled self-assembling helical tube composed of 50 3D-printed capsomeres; (**b**) three-dimensional rendering of an individual capsomere, highlighting the interlocking trapezoidal prongs and lock mechanism.

To model the self-assembly process of a virus like TMV, we 3D-printed identical capsomeres using PLA filament (Figure 2). To design the shape, we used Autodesk’s Fusion 360 CAD software (v.16.7.0.2155) [58] to radially project a helical structure onto a cylinder and then cut the resulting helix into equal-length segments, forming the individual capsomeres. In our model, the pitch p was set to 9 mm, with approximately 5 and 1/3 capsomeres per helical turn. Although simplified, the model captures essential features of the viral self-assembly process. Each capsomere was designed identically, with geometric interaction sites modeled after viral capsids.

To simulate the attractive forces that guide viral self-assembly, we placed small cylindrical magnets in on each end of the 3D-printed capsomeres. These magnets facilitated the alignment and interaction between adjacent subunits, allowing them to self-assemble into stable helical structures as shown in Figure 2a.

A critical element for ensuring the assembly and stability of the 3D-printed capsomeres was the design of interlocking components as seen in Figure 2b. This mechanism serves three key functions in the self-assembly process: (i) preventing disassembly with minimal assembly impedance; (ii) enhancing the rigidity and structure of the formed piece; and (iii) avoiding improper connections. These three things were all accomplished by manipulating the capsomere end geometry. One end of the capsomere is an extruded trapezoid with two extending prongs which act like a key, the other end is the negative complement of the trapezoidal pronged extrusion which acts like a lock. Once connected, the capsomere pieces cannot swivel or hinge apart as the prong nearest to the hinging point collides with the inner wall on the lock side. The tapered geometry on the lock and key features was included to stop improper assembly orientations from occurring. Without the taper inducing mismatching prong sizes, flipping the capsomere 180 degrees would allow for an incorrect connection incapable of coming apart from the tumbling forces. While these geometric features may seem intricate, they are far simpler than the actual capsid proteins found in nature, where much more complex, uneven geometries interlock like puzzle pieces to form stable structures.

## 4. Design of the Dual-Spiral Helical Model

**Figure 3:**
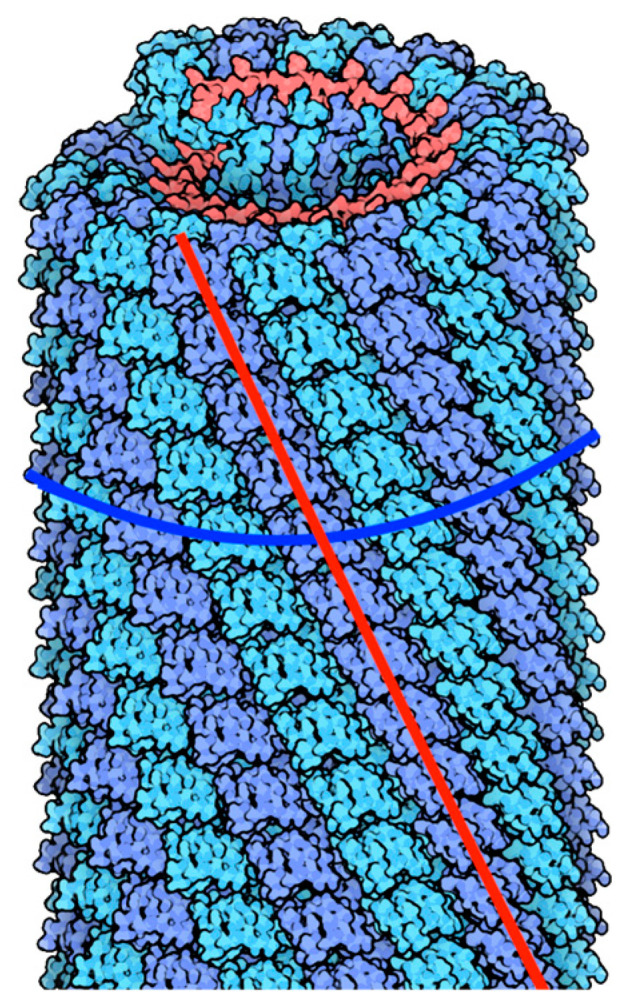
Helical organization in Tobacco Mosaic Virus (TMV). The image highlights left-handed (red curve) and right-handed (blue curve) helices. To aid in visualization the left-handed helices are depicted throughout in blue and purple. The image has been adapted under a Creative Commons license from the Protein Data Bank’s Molecule of the Month (https://pdb101.rcsb.org/motm/109 (accessed on 5 December 2024)).

Our goal in developing self-assembling helical models was to produce structures with more complex geometry by combining left-handed and right-handed helices together into a single model. This choice was motivated by phyllotactic patterns [59,60,61,62,63,64,65,66] that are frequently seen in nature, such those seen in pinecones and sunflowers, where the number of spirals is determined by successive Fibonacci numbers. The pair of opposite spirals in phyllotaxis are referred to as parastichy numbers; e.g., (3,5) in pine cones; (8,13) in pineapples; (21,34) in sunflower heads and, (34,55) in artichokes. Occasionally, numbers in the Lucas series are also observed in such systems. Erickson [60] noted “the protein coats of viruses can be specified by citing the index numbers of two or three sets of contact parastichies, or helical ranks of monomers, as has been done in classical studies of phyllotaxis. This specification has the form k(m, n) or k(m, n, m + n), where m, n, and (m + n) are parastichy numbers specifying screw displacements, and k is the jugacy, or frequency of rotational symmetry. For simple structures, k = 1. This notation has the advantage of terseness and of indicating the basic isometries of these helically symmetrical structures”. Figure 3 illustrates the left-handed and right-handed helices observed in Tobacco Mosaic Virus (TMV). This visual representation highlights the biological basis for the dual-spiral structure modeled in our designs.

**Figure 4:**
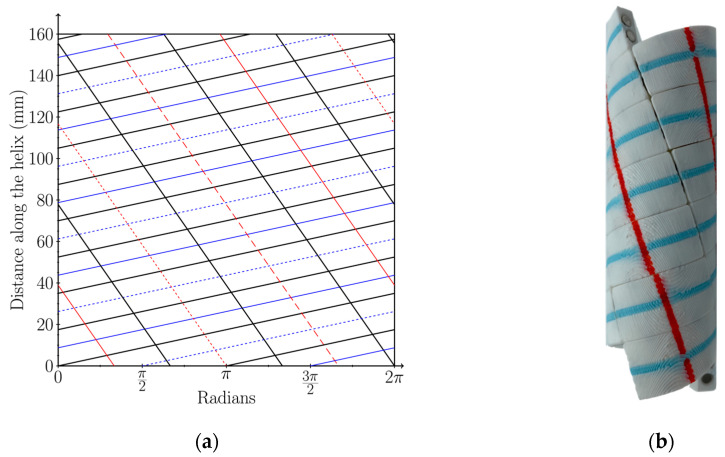
Dual-spiral phyllotactic helical model: (**a**) planar projection of an infinite cylinder with left- and right-handed spiral wrappings with a 2,3 phyllotaxis. The three left-handed helices are represented by the red lines: solid, short-dashed, and long-dashed, while the two right-handed helices are represented by the blue lines: sold and short-dashed; (**b**) self-assembled helical model demonstrating the 2,3 phyllotaxis, where the red and blue lines shown in (**a**) are represented as solid lines on the model.

To develop our dual-spiral helical model, we cut a cylinder into individual capsomeres using two right-handed helices and three left-handed helices, resulting in a dual-spiral structure with 180-degree symmetry. Figure 4a shows the net diagram of this dual-spiral model, where the black lines represent the cutting helices, forming the edges of the components. The right-handed helices in this model are positioned π radians apart around the circumference of the cylinder, while the left-handed helices are separated by π/3 radians. Note that in Figure 4a these helices intersect the x-axis at 0 and π for the right-handed helices and π/3, 2π/3, and 2π for the left-handed ones, resulting in a grid of parallelograms. In Figure 4b, the 3D-printed model highlights these spirals, with red and blue lines running down the middle of each component, clearly showing the structure’s dual-spiral design.

The Fibonacci sequence, defined as:

(4) $$F_{0}=0,\;F_{1}=1,\;F_{n}=F_{n-1}+F_{n-2},\;n\geq 2\;$$

was essential to achieving designs that self-assemble efficiently and also are aesthetically pleasing. Fibonacci spirals optimize packing and growth efficiency in natural forms such as leaves, seeds, and petals.

To achieve a helical cylinder that efficiently self-assembles, it is essential to select a n,m phyllotaxis pattern, where n and m represent the number of spirals in opposing orientations, that optimizes the shape of the 3D-printed capsomere. When prototyping our models we found that capsomeres with varying edge lengths that were not overly elongated provide the best chance for a rapid assembly. For instance, we found that using a n,n phyllotaxis leads to issues as the capsomere’s outer faces form slightly curved rhombi with curvature in one direction but not the other. This complicated the spacing of the magnets along each edge and often resulted in assembly errors, such as pieces joining at incorrect 90-degree angles. These rotated capsomeres do not fit properly along the curvature of the cylinder and would need to be knocked loose before the assembly could continue.

On the other hand, a ratio of n/m that is far from 1, like 2/8, where there are 2 spirals in one orientation and 8 in the other, results in long, skinny parallelograms that tended to form less stable structures. Due to these results and our attempt to model our designs after nature we decided to select n and m to be a pair of consecutive Fibonacci numbers (e.g., 2 and 3). This ensured that our parallelogram-shaped capsomeres have an aspect ratio close to 1 and are neither too square nor too elongated, simplifying the assembly process.

**Figure 5:**
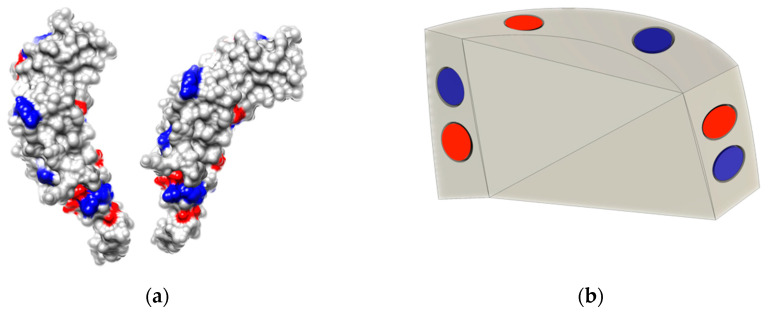
Charge distribution and magnet orientation in capsomeres: (**a**) charged residues on TMV capsomeres, with positive arginine (blue) and negative aspartic acid (red) residues; (**b**) interior of our 3D-printed capsomere model showing magnets aligned to mimic the TMV charge pattern, with north (negative, red) and south (positive, blue) poles.

In comparison to our interlocking helical design discussed in Section 3, the simpler parallelogram design of our dual-spiral model required a more complicated magnet map. To help inform placement and orientation of magnets along our 3D-printed capsomeres we looked to nature once again. Using UCSF Chimera, we visualized the charged residues located on the surface of Tobacco Mosaic Virus (TMV) capsomeres [67]. As shown in Figure 5a, arginine residues with positive charges are colored in blue, while aspartic acid residues which have negative charges are shown in red. By examining the positions of these residues along the protein’s edges we identified a general alternating charge pattern. Furthermore, when focusing on the interaction sites between adjacent capsomere proteins, we observed that each positively charged residue on one protein aligns in close proximity with a negatively charged residue on the neighboring protein. This alignment helps to facilitate the electrostatic attraction between two capsomers, stabilizing the helical structure.

A However, while the number of interaction sites found between two neighboring capsomeres in TMV helps to provide stability of the structure, we found that this pattern was more complex than necessary for the successful self-assembly of our models. Through experimentation it was discovered that a simplified version of the alternating charges was sufficient to facilitate the self-assembly. To approximate the charges found on TMV, we placed eight magnets on each 3D-printed subunit, with two magnets along each interaction edge, alternating north and south poles as shown in Figure 5b. The magnets were aligned with wider spacing on the longer edges compared to the shorter ones, thus ensuring the formation of the components into a singular helical tube.

Although our model was simplified compared to the structure of an actual helical capsid, we conclude that this representation effectively replicates the essential electrostatic forces, ensuring proper alignment and stable assembly through magnetic attraction. While the eight magnetic interaction sites did not capture the full complexity of the charge distribution of TMV, it successfully demonstrated the concept of capsid self-assembly, thus showing that local interactions can drive large-scale organization. This proof of concept confirmed that magnetic forces could model key interactions in helical viral capsid assembly, opening avenues for further exploration and refinement.

## 5. Tumbler Design and Self-Assembly Optimization

In nature, the formation of viral capsids is a dynamic process, where viral protein subunits spontaneously come together through random interactions, forming stable structures with minimal guidance. Our aim in this work was to replicate these random interactions while also providing a repeatable environment that promotes successful self-assembly. To accomplish these goals, we designed a cylindrical tumbler that is constructed from mostly 3D-printed parts that we designed in Fusion 360. The goal of the cylinder was to help guide the assembly process by mimicking the random tumbling and collisions that occur between viral capsomeres inside of a host cell.

**Figure 6:**
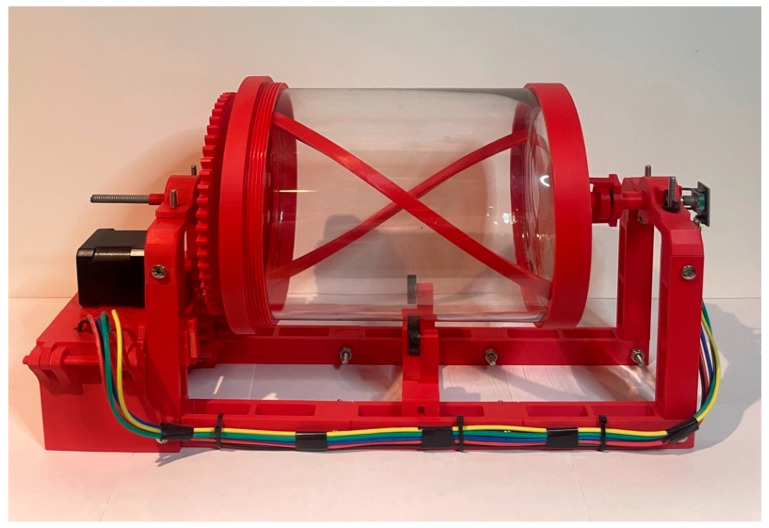
Cylindrical tumbler. Our cylindrical tumbler designed to facilitate random tumbling and self-assembly of capsomeres.

Our tumbler, shown in Figure 6, was designed with five main constraints in mind: (1) low cost, making it accessible for a variety of research and educational applications; (2) high visibility, allowing viewing of the assembly process during the tumbling; (3) a balanced container, for stability while rotating; (4) a randomized tumbling mode, to mimic biologically relevant, random assembly conditions; and (5) adjustable tumbling speed and motion, offering greater control and repeatability compared to hand-mixing the pieces in different containers like spheres, cylinders, and cones.

To facilitate the mixing action and prevent the capsomeres from spreading too far apart during assembly, we added two helical ridges inside the cylinder’s inner circumference, creating an Archimedean screw effect. The combination helical ridge and cylindrical shape of the container helped to keep the capsomeres in constant contact with each other while allowing larger sub-assembled sections to roll along their longitudinal axes. Maintaining the proper alignment of these sections at the bottom of the tumbler was critical to promoting assembly.

To decrease the assembly time of our models we experimented with helical ridges of varying thicknesses. Thicker ridges created more vigorous tossing, which helped to increase the number of interactions between capsomeres. However, when the action was too forceful this frequently resulted in disassembly of large sections of the model. To achieve rapid assembly, we needed a delicate balance between gentle rolling and more forceful actions. To help achieve this the tumbler’s speed was also made adjustable so that we could have better control over the frequency and force of interactions. Adjusting the speed and screw thickness for each model to find the right balance was key to optimizing the self-assembly process.

Interestingly, we found that reducing the number of magnets on each capsomere improved the efficiency of self-assembly. This aligns with the principle that overly complex charge arrangements can lead to random clumping, hindering orderly assembly. By keeping the magnetic configuration simpler, the pieces assembled more effectively under the controlled random motions inside the tumbler.

## 6. Materials and Methods

**Figure 7:**
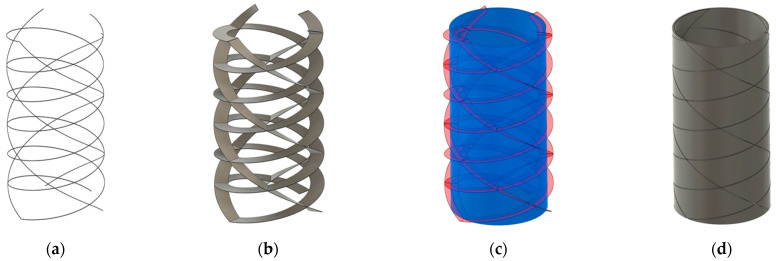
Capsomere design process: (**a**) five intersecting helical paths; (**b**) rectangles swept along paths to form 3D bodies; (**c**) subtraction of helical bodies from cylinder; (**d**) resulting parallelogram-shaped capsomere pieces following a dual-spiral pattern.

The capsomeres for our helical model were designed using Autodesk’s CAD software, Fusion 360 as illustrated in Figure 7. To design the shape of the capsomeres we first created five intersecting helical paths, three left-handed and two right-handed, along the surface of a cylindrical structure. Next, we swept the profile of a thin rectangle along the path of each helix to create two 3D bodies that intersected the cylinder. Using a Boolean difference operation, these bodies were then subtracted from the cylinder, producing individual parallelogram-shaped capsomere pieces that follow the dual-spiral pattern of the intended model. We then radially thickened each piece so that the edges of the models could accommodate the magnets.

To simplify the 3D-printing process, we cut each capsomere so that they had two flat-bottom faces along their inner surface, as shown in Figure 5b. This flat-bottom design allowed the pieces to rest directly on the print bed, eliminating any need for support material while printing. This design choice eliminated any necessary post-processing of the finished 3D-prints and allowed us to go straight from printing the pieces to inserting the magnets. The design of our capsomeres was intentionally kept as simple as possible to help facilitate the reproducibility of our work in an educational environment.

To fabricate each of the capsomeres we utilized a P1S 3D-printer from Bambu labs. All parts of our model and tumbler were printed from Polylactic Acid (PLA) filament. For optimal print quality and speed of the printing process, we used a 0.2 mm layer height. This provided a relatively smooth surface finish suitable for our models and allowed us to print in multiple colors without overly extending the print time. An infill density of 20% was selected to balance durability with material efficiency, yielding capsomeres that were robust enough for handling without excess material usage. The flat-bottom design also allowed for efficient bed adhesion such that we did not require a brim during printing, further improving manufacturing efficiency. Each capsomere was designed to accommodate eight 3 mm diameter neodymium magnets. To account for the tolerance of the P1S printer, we designed the diameter of each magnet hole to be slightly larger than that of the magnets at 3.2 mm. This slight increase in diameter allowed the magnets to be press-fitted into the holes with relatively little force, securely holding them in place.

For added adhesion of the magnets to the capsomeres we decided to add a small amount of glue to each hole in the model. We found that common super glue proved insufficient to hold the magnets in place under the action of our tumbling container. Instead, using Loctite 401, which is designed to have a more durable bond, provided a robust solution that effectively secured each magnet in place, ensuring durability throughout the assembly process.

**Figure 8:**
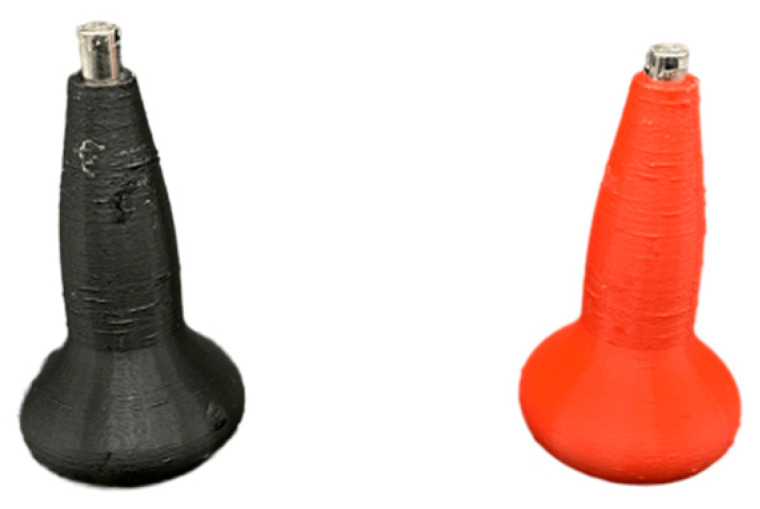
Magnet orientation tools. Custom, color-coded tools to ensure consistent north–south polarity during magnet installation.

To speed up the installation of the magnets and maintain consistent polarity across all capsomeres, we designed a custom, color-coded tool featuring a set of larger 5 mm diameter cylindrical magnets contained in a user-friendly 3D-printed holder. This setup, illustrated in Figure 8, allowed us to pick up each 3mm diameter magnet individually. We then applied a small amount of glue to each hole in the model and carefully pressed a magnet into place, ensuring that the desired north–south polarity was achieved on each edge. This attention to polarity was critical for successful self-assembly in the final helical structure.

## 7. Experimental Results

To verify our methodologies and compare the efficiency of the self-assembly process for each of our models, we conducted time trials using the cylindrical tumbler shown in Figure 6. Each model was tested by placing 15 3D-printed capsomeres into the tumbler and running it at two rotational speeds: 17.7 RPM for 10 trials and 33.2 RPM for an additional 10 trials. These speeds were selected based on prior observations, where the single-helix model assembled most efficiently at the slower speed and the dual-helix at the faster speed. Each trial lasted 5 min, with data recorded at the end of every minute.

We chose to begin each trial with 15 capsomeres to reflect a fully disassembled state. This number was sufficient to form three complete turns of the helix in the single-helix model, providing a balance between observing assembly dynamics and achieving manageable trial durations. While larger models take significantly longer to assemble, this setup allowed us to conduct more trials and ensure repeatability within a reasonable timeframe.

**Figure 9:**
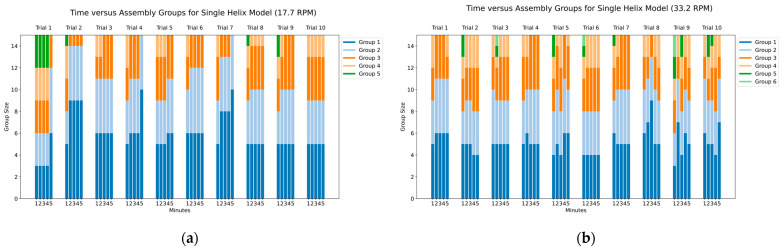
Time versus assembly groups for single-helix model: (**a**) assembly with tumbler at 17.7 RPM; (**b**) assembly with tumbler at 33.2 RPM.

**Figure 10:**
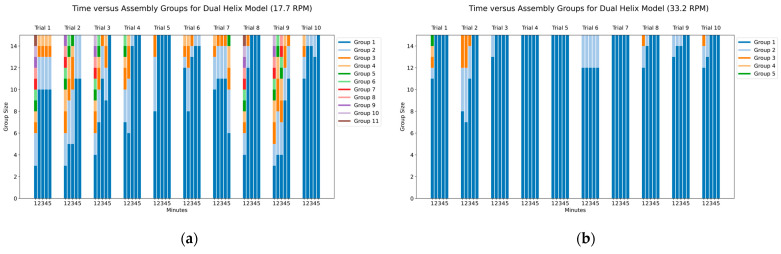
Time versus assembly groups for dual-helix model: (**a**) assembly with tumbler at 17.7 RPM; (**b**) assembly with tumbler at 33.2 RPM.

To effectively capture the assembly process, we tracked both the number of groups formed and the number of capsomeres in each group. As illustrated in Figure 9 and Figure 10, the general trend shows the number of groups decreasing over time as the number of capsomeres within each group grows. In our charts, these groups are represented as stacked bar segments. For example, a single bar with segments of lengths 6, 5, 4 would indicate three groups, one containing six capsomeres, another five, and the last four. A successful complete assembly results in a single group of size 15, represented in the bar chart as a single bar 15 units tall, indicating that all capsomeres have connected into one continuous chain.

The charts in Figure 9 and Figure 10 provide a clear physical representation of the assembly process for each rotational speed and helix model. This visualization offers insight into how efficiently each model assembles under varying conditions, serving as a direct depiction of the assembly state within the tumbler.

The observed behavior of the assembly process for the single-helix model was generally consistent across both rotational speeds, though the slower speed of 17.7 RPM demonstrated slightly better performance. As shown in Figure 9a, the slower rotational rate resulted in a maximum of five groupings of capsomeres across all trials and time points. In contrast, the data for the faster speed, shown in Figure 9b, revealed instances of six groupings in three of the ten trials. At the five-minute mark, the average number of groups was 3 for the slower speed, compared to 3.8 for the faster speed.

Figure 9b also highlights occurrences where larger subassemblies broke apart into smaller groups, a phenomenon not observed at the slower rotational speed depicted in Figure 9a. Instead, at the slower speed, the assembly process was more stable, with groups steadily growing in size over time. This suggests that the higher impact forces associated with the faster rotational speed can disrupt the single magnet interaction sites of the single-helix model, leading to disassembly rather than progression toward complete assembly.

When collecting the assembly data for the dual-helix model, we observed a significant difference in the rates of assembly between the two rotational speeds of the tumbler. The increased rotational speed appeared to facilitate a greater number of interactions between the capsomeres, leading to faster assembly. The dual-helix model demonstrated resilience to the increased force due to its additional magnetic interaction sites between pieces. This resilience allowed the higher number of interactions to result in efficient assembly without disassembly, unlike the single-helix model.

It is important to note that, in a biological context, capsomeres undergo assembly in an environment driven in part by Brownian motion, allowing for far more frequent molecular encounters than in our tumbler model. This natural motion likely accelerates the assembly process, as it does not involve the need to overcome forces such as gravity or impact from the tumbler walls. The dual-helix model’s ability to withstand greater force in our experiment mimics this biological efficiency, where frequent interactions between subunits optimize the assembly process.

Figure 10 illustrates that maximizing the number of interactions while avoiding disassembly dramatically reduces the time required for complete assembly. At the faster rotational speed, Figure 10b shows nine of the ten trials resulted in complete assembly within 5 min, with three trials achieving this in the first minute. In contrast, at the slower speed shown in Figure 10a, only half of the trials achieved complete assembly, with the average number of groups at the 5 min mark being 1.1 for the faster speed compared to 2.1 for the slower speed.

Given the importance of structural stability in our models, we conducted drop tests at various heights to evaluate their robustness. Drops were performed from an initial height of 1 cm, incrementing by 1 cm for each subsequent test. The models were dropped onto a hard surface, ensuring they landed parallel to their main axis to standardize the impact. For each height, we conducted 10 trials for both the single- and dual-helix models. Testing was concluded at the height where all 10 trials for a given model showed some degree of disassembly.

**Figure 11:**
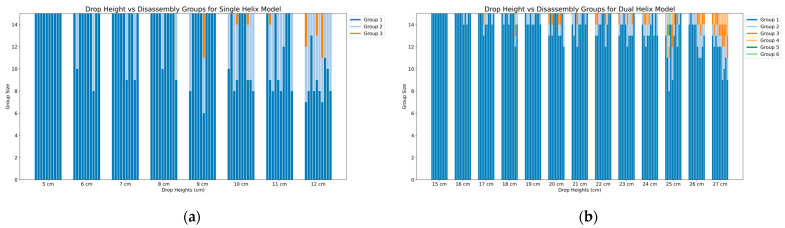
Drop height versus disassembly groups for single- and dual-helix models: (**a**) single-helix model; (**b**) dual-helix model.

Figure 11 provides a visual representation of the drop height against the resulting number and size of disassembled groups for each model. As expected, the general trend shows greater disassembly with increasing drop height. Figure 11a illustrates the results for the single-helix model. No disassembly was observed for drops below 6 cm, but at a height of 12 cm, disassembly occurred in all 10 trials. This behavior aligns with the tumbler data, highlighting the limited structural stability of the single-helix model under conditions of greater impact.

Conversely, the dual-helix model exhibited significantly greater robustness, as shown in Figure 11b. No disassembly was observed for drop heights below 16 cm, and all 10 trials showed disassembly only at the maximum tested height of 27 cm. This increased stability mirrors the model’s performance in the tumbler, where the additional magnetic interaction sites allowed it to withstand higher forces without disassembling.

Together, these results emphasize the relationship between structural design and stability under both controlled assembly conditions and external impact, highlighting the dual-helix model’s superior resilience and assembly efficiency. More data can be found in the Supplementary Materials.

## 8. Conclusions

The 3D-printed helical capsid models we present here offer an effective approach for exploring viral self-assembly at the mesoscale. By integrating the geometry of a 3D-printed helical component with simplified magnetic interactions, we were able to achieve the reliable assembly of helical structures that closely mimic the principles underlying viral capsid formation. This research illustrates that the self-assembly of helical structures which are modeled after the complex geometries and/or charge interactions of viral capsids is possible, providing new insights into the mechanics of helical self-assembly.

Future work in this area could research the assembly times and robustness of these models while adjusting a few additional variables. Namely, one could investigate the role that different phyllotactic ratios, magnet configurations, and infill densities have on the speed of assembly and structural stability of the models. In addition, adjusting the forces between components by using differently sized magnets could be used to further explore the impact of charges on the assembly. While a more complicated endeavor, attempting to more closely match the exact geometry of a viral capsomere such as TMV could be explored. This could include models mimicking the action of capsomeres tethering to RNA, which serves as a guiding factor in the assembly process. This additional research would further refine our understanding of the dynamics that drive self-assembly, enhancing these models’ resemblance to biological capsid formation.

Our approach here demonstrates how versatile 3D-printing is for producing intricate self-assembling models that may be used in reach and teaching environments. These models are useful tools in molecular biology, nanotechnology, and material science as they provide a concrete way to visualize viral architecture, phyllotactic patterns, and symmetry in a hands-on way. Their reproducibility promotes experiential learning, closing the gap between theoretical understanding and real-world investigations, increasing scientific engagement and understanding.

## Data Availability

Plante, D., Unzen, K, Jungck, J. R. (2024). 3D-Printed Self-Assembling Helical Models: Assembly Data, Stability Tests, and STL Files. QUBES Educational Resources: doi:10.25334/45JS-8842.

## Supplementary Materials

The following supporting information can be downloaded at: https://www.mdpi.com/article/10.3390/biomimetics9120763/s1. We have available videos of the models assembling in the tumbler. The plans for the tumbler are available at: https://skfb.ly/oKFYo, accessed on 7 March 2024.
