# Supplementary figures and images for: 3D-Printed Self-Assembling Helical Models for Exploring Viral Capsid Structures
Source: Biomimetics (Basel). 2024 Dec 16;9(12):763. doi: 10.3390/biomimetics9120763 (PMC11673919; doi:10.3390/biomimetics9120763)

## Tumbler Electrical Schematic

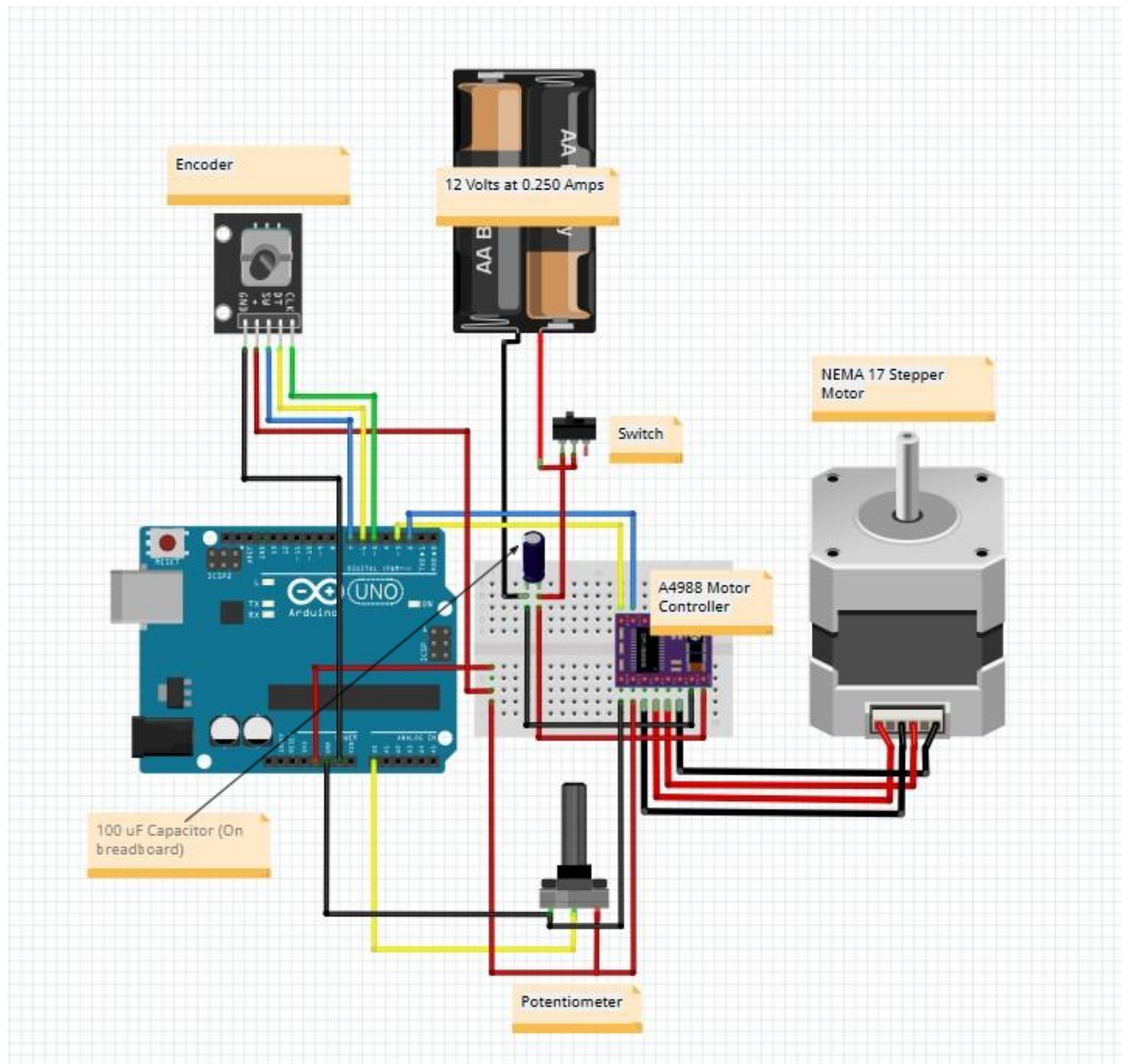

3D Viewer of Tumbler Model With Notes:

<https://skfb.ly/oKFYo>

Supplement: Supplementary file 1 [file biomimetics-09-00763-s001.zip › biomimetics-3342254-supplementary.pdf]
